# Risk Characterization and Benefit–Risk Assessment of Brominated Flame Retardant in Commercially Exploited Freshwater Fishes and Crayfish of Lake Trasimeno, Italy

**DOI:** 10.3390/ijerph18168763

**Published:** 2021-08-19

**Authors:** Rossana Roila, Raffaella Branciari, David Ranucci, Arianna Stramenga, Tamara Tavoloni, Tommaso Stecconi, Raffaella Franceschini, Arianna Piersanti

**Affiliations:** 1 Department of Veterinary Medicine, University of Perugia, Via San Costanzo 4, 06126 Perugia, Italy; rossana.roila@unipg.it; 2 Istituto Zooprofilattico Sperimentale dell’Umbria e delle Marche “Togo Rosati”, Via Cupa di Posatora 3, 60131 Ancona, Italy; a.stramenga@izsum.it (A.S.); t.tavoloni@izsum.it (T.T.); t.stecconi@izsum.it (T.S.); a.piersanti@izsum.it (A.P.); 3 Department of Engineering Sciences, Guglielmo Marconi University, 00193 Rome, Italy; r.franceschini@unimarconi.it

**Keywords:** flame retardants, MOE, exposure assessment, risk characterization, benefit characterization, fish products, freshwater fish, benefit–risk assessment

## Abstract

Among brominated flame retardants (BFRs), polybrominateddiphenyl ethers (PBDEs) and hexabromocyclododecanes (HBCDs) were the most widely used in past decades. BFRs not being chemically bonded to polymers means they can easily leach from the products into the environment and bioaccumulate. Humans are exposed to flame retardants mainly through food consumption, especially fish and fish products. In the present study, the occurrence of PBDEs and HBCDs in freshwater fishes and crayfish from Lake Trasimeno (Umbria region, central Italy) was assessed according to monitoring plans recommended by European competent authorities. The dietary exposure of the central Italian population to such molecules was calculated, and the risk characterization and the benefit–risk evaluation were also assessed. A total of 90 samples were analyzed by means of gas and liquid chromatography associated with triple quadrupole mass spectroscopy. A total of 51% of samples were found positive for at least one of the congeners; the most frequently found molecule was BDE-47. The data on dietary exposure ranged from 0.138 to 1.113 pg/kg body weight/day for ∑PBDE and from 0.805 to 0.868 pg/kg body weight/day for ∑HBCD. The data show no health risks for the central Italian population consuming freshwater fish products from Lake Trasimeno in relation to exposure to PBDE and HBCD.

## 1. Introduction

The term flame retardants (FRs) refers to a diverse group of chemical compounds which are added to manufactured materials, such as plastics, textiles, circuitry and building materials, in order to prevent or delay flames [1]. One of the most important classes of these compounds is represented by halogenated flame retardants (HFRs), particularly the subgroup of brominated flame retardants (BFRs). 

Since the early 1970s, the most widely used BFRs have been polybrominated diphenyl ethers (PBDEs), a class of 209 congeners which differ in the number and position of the bromine atoms in the two phenyl rings and are commercialized as three technical mixtures characterized by different bromination degrees: penta-BDE, octa-BDE and deca-BDE [2,3]. As they are mixed into polymers and not chemically bound, they might separate or leach from the products into the environment, and they have already been demonstrated to be environmental contaminants several years after their technological application [2]. Due to their potential adverse health effects and other factors such as their resistance to degradation, their persistence in the environment, their widespread global distribution and their ability to bioaccumulate and biomagnify in the food chain, international agreements on the regulation and use of some PBDEs have been introduced since 2003 [3,4]. Penta- and octa-mixtures were banned in the European Union (EU) in 2003, while, since 2008, deca-BDE can no longer be used in electronics and electrical applications [5,6]. In 2009, penta- and octa-BDE were included in the Stockholm Convention elimination list (Annex A), followed by deca-BDE in 2017 [7,8,9]. Similar to PBDEs, hexabromocyclododecanes (HBCDs) represent an important and widely used group of BFRs [10] mainly applied in construction and packing material [11]. HBCDs comprise 16 stereoisomers; however, technical products primarily include three isomers (α-, β- and γ-HBCD), of which the most relevant is γ-HBCD followed by α- and β-HBCD. As already mentioned for PBDEs, HBCDs are mixed into polymers and not chemically bound to plastic or textiles; therefore, the release of this chemical into the environment is highly feasible [12]. Considering HBCDs’ persistence, low water solubility, high octanol–water partition coefficient and toxic effects, in 2008 they were classified by the European Commission (EC) as bioaccumulative and toxic compounds [13]. The United Nations Stockholm Convention listed HBCDs as Persistent Organic Pollutants (POPs) in 2013 [14], and their use has been phased out in many fields by 2016 [15]. 

Despite the advances in the prevention of fire incidents reached by using FRs, the prolonged, extensive and frequently unrestricted use of these compounds in the past has resulted in their ubiquitous diffusion in various environmental matrices [16,17]. As a consequence, humans can be exposed to both PBDEs and HBCDs through numerous routes such as inhalation, dust ingestion and food and water consumption [3]. Although the precise contribution of these sources is significantly influenced by specific characteristics of compounds, populations and even individuals, there is a common consensus in the scientific community that food consumption appears to be the major source of exposure to such chemicals for the general population [2,11,15,18,19]. The European Food Safety Authority (EFSA) released two scientific opinions on PBDEs and HBCDDs assessing their occurrence in food, related human exposure and the associated risk [2,11]. The opinions were structured based on data provided by EU Member States; however, Italy did not participate in the call for data promoted by the Authority. The EFSA panel on contaminants in the food chain suggested that both molecule groups are characterized by low acute toxicity; however, chronic toxic effects have been reported [2,11]. 

PBDEs can cause hepatocellular hypertrophy, developmental and reproductive impairments, perturbation of thyroid hormone regulation and fetotoxic effects [2]. Similarly, major targets for chronic toxicity of HBCDs are the liver, thyroid hormone homeostasis and the reproductive, nervous and immune systems [11]. Higher levels of contamination have been attributed to products of animal origin, and, specifically, as a result of the combination of contamination level and food consumption, the category “Fish and other seafood” has been identified as the main contributor to human dietary exposure [2,11]. In light of the persisting use of products containing such chemicals, the European Commission in 2014 issued the Recommendation 2014/118/EU encouraging the competent authorities of Member States to monitor BFRs in food with the aim of including a wide variety of foodstuffs reflecting the consumption habits to achieve an accurate estimation of exposure in different food commodities [20]. It is recommended that the surveillance of PBDEs and HBCDs operated by competent authorities should continue and that further epidemiological studies based on thorough estimates of human exposure should be promoted [11].

In this context, the present study aimed to define the dietary exposure to PBDEs and HBCDs of a population in central Italy in relation to the consumption of freshwater fishes and crayfish caught in Lake Trasimeno (Umbria region, central Italy). The consumers’ risk related to dietary exposure to such molecules was characterized, and a benefit–risk evaluation associated with the consumption of these selected food products was also performed.

## 2. Materials and Methods

### 2.1. Sampling

Lake Trasimeno is one of the largest lakes of the Italian peninsula, characterized by a large surface area of about 128 km^2^, but it is quite shallow with a maximum depth of 6 m (Figure S1). Lake Trasimeno is very rich in fish, and its fish fauna comprises 19 species dominated by those belonging to the Cyprinidae family [21]. Traditional fishing is one of the main commercial activities of the lacustrine area, promoted by governmental guidelines encouraging the production and consumption of Km 0 food. In this context, the most represented edible fishes and crayfish captured and consequently consumed were analyzed for safety aspects linked to the presence of specific classes of POPs: carp (*Cyprinus carpio*, L., average length 75.03 ± 13 cm, esteemed age 3–6 years old), goldfish (*Carassius auratus*, L., average length 25.34 ± 2.04 cm, esteemed age 3–4 years old), tench (*Tinca tinca*, L., average length 39.87 ± 2.54 cm, esteemed age 1–2 years old), eel (*Anguilla anguilla*, L., average length 55.23 ± 8.76 cm, esteemed age 4–6 years old), perch (*Perca fluviatilis*, L., average length 19.92 ± 1.24 cm esteemed age 1–1.5 years old), and crayfish (*Procambarus clarkii*, G., average length 7.75 ± 4.20 cm, esteemed age from 6 months to 1 year old). Fishes and crayfish were collected within the framework of the official monitoring program during the years 2018–2021 according to the EC Regulation 2017/644 [22]. 

### 2.2. Chemical Analysis

The method applied for brominated flame retardant has already been described by Tavoloni et al. [3], and it is an isotopic dilution analysis. Briefly, 20 g of sample was weighed in a polypropylene centrifuge tube, spiked at 1 ng/g with labelled internal standards and submitted to QuEChERS extraction. Upon extraction, 10 mL of the upper organic layer was transferred into a clean glass tube and reduced in volume at 35 °C using the Genevac EZ-2 concentrator (SP Scientific, Ipswich, Suffolk, UK). The obtained residue was submitted to clean-up on acidic Extrelut NT-3/SPE Si 1 g/6 mL chromatographic assembly and gel permeation chromatography (Gilson GPC system equipped with ASPEC XL auto sampler, 307 HPLC pump and UV-vis detector; Gilson, Middleton, WI, USA). The GPC collected eluent was equally divided into two fractions and reduced to dryness. One fraction was analyzed for PBDEs and the second for HBCDs.

PBDE analysis was conducted in GC-QqQ-MS/MS (7890A GC–7000B MS; Agilent Technologies, Palo Alto, CA, USA) using large volume injection (PTV inlet), and chromatographic separation was achieved on a RTX1614 column (15 m × 250 μm × 0.10 μm; Restek) using He as carrier gas. HBCDs, on the other hand, were analyzed by LC- QqQ-MS/MS (1200 HPLC, Agilent Palo Alto, CA, USA; 3200 Q TRAP; AB Sciex, Darmstadt, Germany), and the chromatographic separation was achieved on a Kinetex XB-C18 column (2.6 μm 100 Å, 100 × 2.10 mm; Phenomenex, Torrance, CA, USA).

PBDE and HBCD are prone to background contamination; therefore, during the batch-to-batch on-going performances assessment, two procedural blanks, a blank sample and the same blank sample spiked at 100 pg/g for PBDEs and 50 pg/g for HBCDs, were processed [3]. External quality assurance was guaranteed by regular participation in inter-calibration exercises organized by the European Union Reference Laboratory (EURL) for halogenated POPs in Feed and Food [23]. LOQs for all the analytes were equal to 10 pg/g except for BDE 206 and 209, which were of 100 pg/g. Analytical determinations were performed on single individuals for all the targeted species except for crayfish, which was considered as a pool (10 specimens per pool).

The left-censored data (results < LOQ) were handled applying the substitution method, as suggested in the literature for studies in the field of food safety [24,25]. The lower bound (LB) and upper bound (UB) approach should be used for ubiquitous chemicals likely to be present in food [24]. The LB was obtained by assigning a value of zero to all samples reported as <LOQ, while the UB was obtained by assigning the numerical value of the LOQ to values reported as <LOQ.

The lipid content of fish flesh and EPA and DHA content were determined according to the procedure of Branciari et al. [21,26]. 

Briefly, EPA and DHA were quantified and expressed in mg/100 g food using the following equation: EPA or DHA (mg/100 g food) = [(AX × WIS × CRFx × CNFx)/(AIS × Ws)] × 1000 × WL, where AX is the EPA or DHA area, AIS is the internal standard area, CRFx is the theoretical correction factor for EPA and DHA, CNFx is the conversion factor from FAME to the corresponding fatty acid (EPA and DHA), WIS is the weight of the internal standard (Methyl nonadecanoate Sigma-Aldrich, Bellefonte, PA, USA) added to the lipids, Ws is the weight of the derivatized lipids and WL is the percentage of sample lipid.

### 2.3. Dietary Exposure Assessment 

The dietary exposure was evaluated through the definition of the estimated daily intake (EDI) of PBDE and HBCD flame retardants [27].

In this scope, EDI was obtained by multiplying the LB and UB concentration of such molecules in food by the amount of fish consumed daily by an average adult weighing 70 kg [27,28]. A questionnaire-based dietary survey was conducted with inhabitants around Lake Trasimeno by randomly selecting and surveying 325 healthy people from the general population. All the participants were local residents, with ages ranging from 19 to 65 years. The questionnaire was designed to obtain information about the frequency of consumption of different freshwater fishes, and the responses were combined with the food portion size data reported by the Italian dietary surveys [28]. The assessors provided their consent prior to the tests; they did not receive any incentives for their participation, and the questionnaires were returned anonymously. No ethical approval was required. 

### 2.4. Risk Characterization

In order to quantitatively estimate the severity of potential adverse health effects in the given population, the risk characterization of PBDEs and HBCDs was performed by means of the margin of exposure (MOE) approach as reported in the literature [2,11,29]. The MOE was calculated by comparing the LB and UB estimated dietary intake for the targeted molecules with the chronic human intake associated with the body burden at the benchmark dose lower confidence limit for a benchmark response of 10% (BMDL_10_) for neurodevelopmental effects in mice, identified as the critical endpoint for some PBDEs [2]. The EFSA panel of experts in a recent reevaluation of the risk assessment of HBCDs in food concluded that, due to some limitations in the assessment, the endpoint for changes in spontaneous behavior in mice (LOAEL of 0.9 mg/kg b.w.) was not suitable for the establishment of a reference point; therefore, a BMDL_10_ was not defined, and the LOAEL was used to define the chronic human dietary intake [11]. 

The chronic human dietary intake (D_r,h_), which reflects the steady state body burden at the calculated BMDL_10_ or at the LOAEL, considering the fraction of the daily intake that absorbed and the body constant rate of the elimination of the compounds [2,11], was used for the calculation of the MOE values according to the Equation (1)

(1) $$\mathrm{MOE}=\frac{D_{r,h}}{\mathrm{EDI}}$$

where MOE—margin of exposure;

D_r,h_—the chronic human dietary intake (µg kg—1 b.w.);

EDI—estimated dietary intake (µg kg—1 b.w.).

According to EFSA, relevant toxicity data were available only for BDE-47, -99, -153 and -209; therefore, in the present study, the risk assessment was carried out exclusively for these four PBDE congeners [2]. Body burdens at the BMDL_10_ of 0.172, 0.0042 and 0.0096 µg/kg b.w./day for BDE-47, -99 and -153, respectively, were considered. In contrast to the other PBDE, for BDE-209, the BMDL_10_ of 1700 µg/kg b.w./day expressed as an external dose can be compared with the estimated human dietary exposure [2]. Concerning HBCDs (considered as the sum of α-, β- and γ-HBCD), the chronic human dietary intake of 2.35 µg/kg b.w./day was used for the risk characterization [11]. The calculated MOE values were then compared to the reference values proposed by EFSA, where an MOE above 24 for HBCDs and above 2.5 for PBDEs-47, -99, -153 and -209 indicates a low health concern, with the risk decreasing as the MOE increases [2,11].

### 2.5. Benefit–Risk Assessment

Beneficial and adverse effects may occur simultaneously in a specific food item within the same range of dietary intake. In order to weigh the benefits and risks associated with food consumption, they should be evaluated and expressed in a comparable way in accordance with the benefit–risk assessment (BRA) paradigm [30].

In the present study, the benefit assessment of fish consumption refers mainly to the ingestion of omega-3 fatty acids, specifically eicosapentaenoic acid (EPA) and docosahexaenoic acid (DHA), identified as active factors in cardiovascular disease prevention [31]; risk factors were attributed to the ingestion of PBDEs and HBCDs, which have been proven to be severely toxic to humans [2,11]. 

Aiming to perform a quantitative estimation of the health benefits of Lake Trasimeno fish consumption, the EPA and DHA content of these fishes was determined analytically as above mentioned, and the exposure assessment of such nutrients in the target population was performed as mentioned above for toxicologically relevant contaminants (Figure S2). Subsequently, the characterization of benefits was determined as the contribution of the exposure values to the attainment of the suggested recommended dietary intake (RDI) of 250 mg/die for EPA and DHA [31].

The benefit–risk quotient (BRQ) was applied to integrate benefit and risk assessment outcomes for the simultaneous ingestion of omega-3 fatty acids and contaminants through freshwater fish consumption, as reported in the literature [32,33]:

(2) $$\mathrm{BRQ}=\frac{Q_{\mathrm{FA}}}{Q_{T}}$$

where Q_FA_ is defined as follows:

(3) $$Q_{\mathrm{FA}}=\frac{R_{\mathrm{FA}}}{C_{\mathrm{FA}}}$$

where R_FA_ (mg/day) is the recommended dietary intake of EPA + DHA. In this study, the RDI of 250 mg/d for a healthy adult [31] was applied; C_FA_ (mg/g) represents the concentration of EPA + DHA in fish muscles. The maximum allowable fish consumption related to toxic effects (Q_T_) can be defined according to the following equation:

(4) $$Q_{T}=\frac{\mathrm{RfD}*b.w.}{c}$$

where RfD (mg/kg b.w./day) is the reference dose of the chemical considered; b.w. is the standard bodyweight set, as mentioned above, at 70 kg; and c (mg/g) is the concentration of each toxic molecule in the targeted fish muscle. The values of RfD considered for the definition of Q_T_ were 100 ng/kg b.w./day for BDE-47 and -99, 200 ng/kg b.w./day for BDE-152, 7000 ng/kg b.w./day for BDE-209 and 200 ng/kg b.w./day for HBCDs [34,35,36,37,38].

BRQ values below 1 suggest that achieving the recommended intake of EPA + DHA poses no evident risk to human health linked to the intake of flame retardants through fish consumption [32,33]. 

### 2.6. Comparative Assessment of Different Contamination Patterns

A comparative assessment was performed to evaluate different contamination patterns related to aquatic environments situated in the same geographical area (Umbria region, central Italy) but characterized by different conformation, ecology and pollution pressure. For this purpose, samples of the same species inhabiting two different waterbodies were considered. A total of 10 samples of perch (*Perca fluviatilis*) collected from lake Piediluco (Umbria region) were analyzed for PBDEs and HBCDs contamination as well as for EPA and DHA content by means of the above-mentioned analytical methods. The risk characterization and benefit–risk assessment were performed, and the results were compared to those of Lake Trasimeno in order to assess the impact of the pollution pressure in relation to the specific habits of the fish species.

## 3. Results

**Figure 1:**
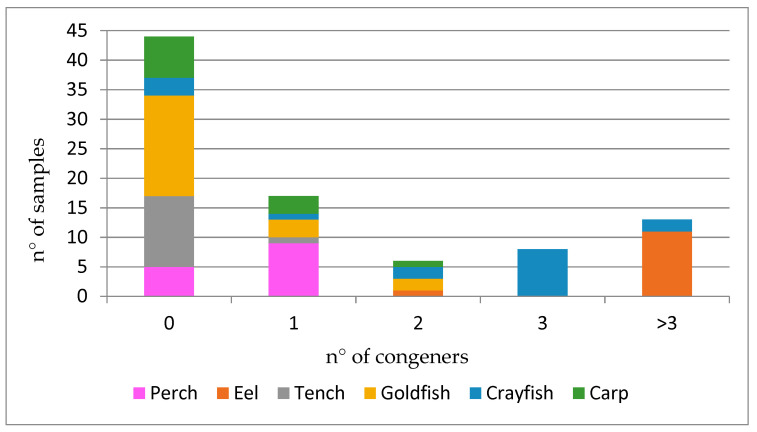
Number and presence of PBDEs and HBCDs in fish species.

A total of 74 freshwater fishes and 16 crayfish pools were analyzed for fifteen BDE congeners (BDE-28, -47,-49, -66, -77, -85, -99, -100, -138, -153, -154, -183, -197, -206, -209) and for three HBCD isomers (α-, β-, γ- HBCD). BFRs were detected in 46 of the 90 (51%) samples analyzed: 17 (19% of the total) were contaminated with one analyte (involving mainly perch) and 29 (32%) with more than one; samples below the LOQ for all BDE and HBCD were 44 (49% of all samples), represented mostly by goldfish and tench (Figure 1). 

**Figure 2:**
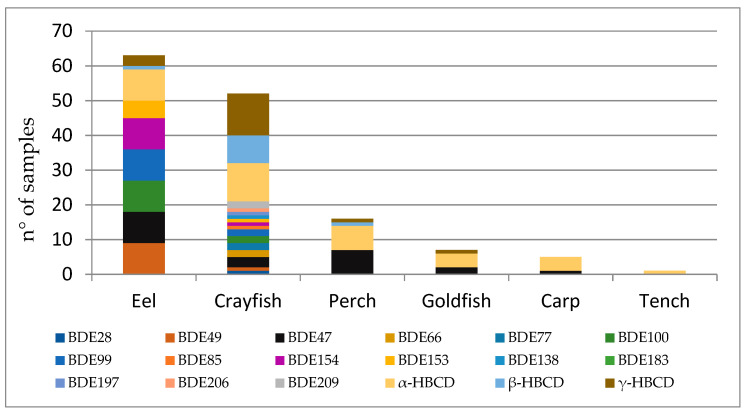
Congeners’ representativeness among different species.

Figure 2 shows the BDE congeners’ and HBCD isomers’ representativeness among different species. Eel was the one with the highest incidence of detected samples (n = 63), mostly due to BDE-47,-49, -100, -99, -154 and α-HBCD (n= 9, all). In crayfish, HBCDs were measured above LOQ in 12 samples with the following pattern: 12, 11 and 8 for γ-, α- and β- HBCD, respectively. PBDEs in crayfish were detected in 3 samples of 16 analyzed, and it is worth noting that one of these was detected with all PBDE with the exception of BDE-183, which was not found in any sample of freshwater fish or crayfish of Lake Trasimeno. Perch is the third species with a larger number of quantified contaminants, mainly BDE-47 and α-HBCD (n = 7, both). Goldfish, carp and tench overall registered less than 10 BRFs analyzed in this study, with the following contamination pattern: α-HBCD was detected in four samples of goldfish, in four carp samples and one tench sample, while BDE-47 was quantified above the LOQ in two samples of goldfish and one sample of carp. Overall, the BDEs’ representativeness among samples is characterized by the following pattern: BDE-47 > BDE-99, -100 > BDE-49, -154 > BDE-153 > BDE-28, -66, -77, -85, -197, -207, -209 > BDE-138. Concerning HBCDs, the dominant was α-HBCD, followed by -γ and –β.

The average PBDEs’ and HBCDs’ LB and UB concentrations in each species considered are shown in Table 1 (detailed data are shown in Table S1a,b). The concentrations of PBDEs and HBCDs are expressed in pg/g wet weight (w.w.); values of ∑PBDE and ∑HBCDs are also expressed in pg/g l.w. (lipid weight) for a better comparison of obtained data with similar results. The ∑PBDE calculated in LB and UB mode was 5.11 and 330.11 pg/g for perch, 437.15 and 718.82 pg/g for eel, 0.00 and 330.00 pg/g for tench, 0.99 and 330.26 pg/g for goldfish, 36.63 and 341.21 pg/g for crayfish and 1.73 and 330.82 pg/g for carp, for LB and UB, respectively. Concerning ∑HBCD, the values ranged from 4.73 to 30.94 pg/g for perch, 730.67 to 747.33 pg/g for eel, 1.24 to 30.56 pg/g for tench, 2.70 to 26.17 pg/g for goldfish, 849.20 to 965.98 pg/g for crayfish and 11.56 to 37.92 pg/g for carp, for LB and UB, respectively.

The food consumption survey shows that the average consumption of freshwater fish species for average adult consumers was 0.47 g/kg b.w./day.

In Table 2, detailed EDIs are reported. The exposure assessment revealed a low intake of BDEs with total values of 0.229 pg/kg b.w./day (ranging from 0.000 to 0.206 pg/kg b.w./day) and of 1.113 pg/kg b.w./day (ranging from 0.146 to 0.339 pg/kg b.w./day), for LB and UB, respectively. The highest value is reached for eel both in LB and UB, and the largest contribution was from BDE-47 (0.112 pg/kg b.w./day). For the other investigated species, the highest contribution to total EDI is given by the BDE-209 UB values, as a result of the higher LOQ attributed to this congener and used in the substitution method for handling left-censored data. On average, the main contributor to the PBDEs’ EDI was BDE-47, followed by -209 and -100, corresponding to 32%, 15% and 14% of the total PBDE intake, respectively. Concerning the four toxicologically relevant PBDEs, besides BDE-47 and -209, -99 and -153 contribute on average to 3% and 2% of the total PBDE EDI, respectively. The total intake of these specific congeners is 0.138 and 0.482 pg/kg b.w./day for LB and UB, respectively.

Concerning HBCDs, the total EDI was 0.805 pg/kg b.w./day (ranging from 0.001 to 0.344 pg/kg b.w./day) and 0.868 pg/kg b.w./day (ranging from 0.014 to 0.455 pg/kg b.w./day) for LB and UB, respectively. The highest value recorded is attributable to eel for α-HBCD LB and UB (0.337 pg/kg b.w./day). Additionally, for the other species, α-HBCD represented the highest of the three isomers with the exception of crayfish, which showed relevant levels of γ-HBCD (Table 2). The main contributor to the HBCD EDI is represented by α-HBCD (53%), followed by γ-HBCD (39%).

In the present study, the risk characterization of toxicologically relevant PBDE congeners and ∑HBCDs was performed by means of the MOE approach by comparing the minimum LB and maximum UB dietary intake for the different molecules with the estimated human intake associated with the body burden at the BMDL_10_, in accordance with EFSA scientific opinions [2,11]. The MOEs ranged from 594 to more than 38,000,000 for the PBDE congeners and between 5160 and over 4,000,000 for HBCDs (Table S2). These MOEs are 2.5 and 24 larger, respectively, for PBDEs and HBCDs; therefore, according to the EFSA CONTAM Panel, they do not raise a health concern [2,11].

The beneficial and adverse effects that may occur simultaneously in the selected freshwater food matrices have been weighted in a comparable way, in accordance with the benefit–risk assessment [30]. The benefit–risk quotient determined in the present study was below 0.0 for all PBDE and HBCD isomers considered (Table S3), attesting that the health benefits related to the consumption of fishery products from Lake Trasimeno outweigh the risks to the consumers. 

The comparative assessment of different contamination patterns through the study of the same species from different water bodies (Lake Piediluco and Lake Trasimeno) showed in perch from Lake Piediluco average LB values of PBDEs ranging from 0 to 130 pg/g w.w. and of HBCDs from 0 to 748 pg/g w.w. Average UB values ranged from 10 to 130 pg/g w.w. and from 12 to 748 pg/g w.w. for PBDE and HBCD, respectively. The most represented PBDE was -47 followed by -99. The exposure values to toxicologically relevant congeners ranged from 0.001 to 0.373 pg/kg b.w./day for LB values and from 0.001 to 0.378 pg/kg b.w./day for UB values (Table S4). 

**Figure 3:**
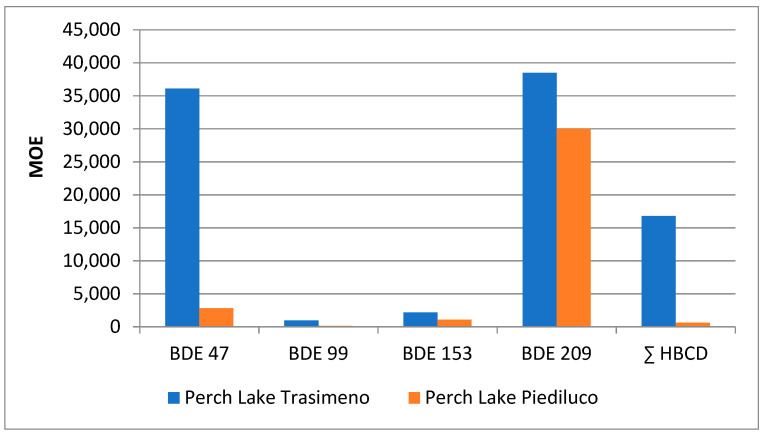
Comparison between MOE values related to perch samples from Lakes Trasimeno and Piediluco.

**Figure 4:**
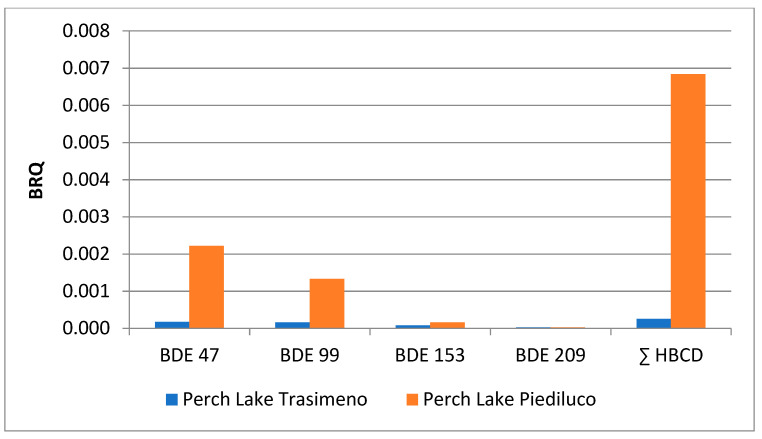
Comparison between BRQ values from perch samples from Trasimeno and Piediluco lakes.

Figure 3 and Figure 4 focus on the comparison assessment between perch samples from the two water bodies, as far as the risk characterization and the benefit–risk quotient are concerned. As shown, although MOE and BRQ for fishes from both lakes are within the range of low concern from a public heath point of view, the data revealed two different scenarios. In particular, the MOE values for Piediluco perch are lower than those of Trasimeno for all the analytes investigated, especially for BDE-47 and ∑HBCDs. In addition, BRQ values are higher for all the toxicologically relevant contaminants.

Values for BDE 209 have been divided by a factor of 1000 and those of ∑HBCD by 10 to allow for a correct interpretation of the graphic.

## 4. Discussion

Despite the ubiquitous distribution of PBDEs and HBCDs, the results of their occurrence in edible species from Lake Trasimeno were characterized by a high proportion of non-detects in accordance with that reported in the literature [2,11]. As mentioned, the overall concentration of HBCDs was slightly higher than the concentrations of PBDEs. Similar results have been found by other authors, which would indicate different HBCDs’ distribution patterns and a more widespread use compared to PBDEs [39]. This evidence may also be associated with the several regulatory restrictions taken for both BFR classes within a different timescale, which reflects biota accumulation [40].

PBDE and HBCD concentrations found in the present study are similar or lower than those observed in previous studies in freshwater species from different countries, albeit the comparison of contamination levels could be difficult due to the different number and type of congeners and isomers analyzed. For eel, Malarvannan et al. [39] in Belgium found a median value for total PBDEs of 60 ng/g l.w. (lipid weight), ranging between 12 and 1400 ng/g l.w., and for HBCDs of 100 ng/g l.w., ranging between 7 and 9500 ng/g l.w. Similarly, Bragigand et al. [41] analyzed PBDEs in eels from Seine and Loire rivers in France with a concentration ranging between 26 and 108 ng/g l.w. Van Leeuwen and de Boer [42] registered in eel samples levels of ΣPBDEs from 3 to 3139 ng/g l.w. In the same species, Roosens et al. [43] in Flanders (Belgium) registered remarkably high levels of PBDEs (660–11,500 ng/g l.w.) and of HBCD (90–12,100 ng/g l.w.), leading the authors to assume the presence of a local source of contamination.

The characteristic biology of eels as bottom dwelling predators with high body fat makes this fish particularly vulnerable to chemical pollution [43]. For instance, due to their specific predisposition for accumulating xenobiotics, the composition of chemical contamination in eels is often interpreted as the result of the local environment pollution pressure serving as a bioindicator. However, it is important to highlight that several factors affect the accumulation of contaminants in eels, such as individual lipid contents and migratory patterns [44]. Some studies note that the level of lipophilic bioaccumulating contaminants in eels is mainly influenced by uptake during their continental growth phase [44]. Furthermore, some authors have hypothesized that brominated and chlorinated flame retardant presence in juvenile eels can be caused by maternal transfer to offspring due to redistribution to the gonads and eggs during maturation [45].

For common carp harvested in Chinese freshwater basins, ∑PBDEs have been found in concentrations of 19.78 ng/kg l.w. in 2009, 16.48 ng/kg l.w. in 2010 and 5.54 ng/kg l.w. in 2011 [46]. In the same study, the levels of ∑HBCDs were 51.9 ng/kg l.w. in 2009 and from 23.7 to 169.6 ng/kg l.w. in 2011. 

In crucian carp from south China, the registered ∑PBDEs were 1430 ng/kg l.w. [47]. Much lower levels of ∑PBDEs contamination were reported in the same year in a study [48] of gibel carp samples from the Danube delta, Romania (2.73 ng/kg l.w.), and in mud carp from south China according to Zhang et al. [47] (10.1 ng/kg l.w.).

Concerning perch from Czech rivers, in a study by Hajslova et al. [49], the amount of ∑PBDEs and ∑HBCDs detected was 3.8 and 3.9 ng/kg l.w., respectively. Covaci et al. [48] also reported a low value for tench of <0.1 ng/kg l.w., in line with what has been observed in present study. In the Danube River, Harrad et al. [49] found a level of ∑HBCD of 0.90 ng/g w.w. for perch, while a maximum of 0.84 ng/g w.w. for α-HBCD for the same species inhabiting the Danube River was reported elsewhere [50]. Concerning tench, an average value of 88.2 ng/g w.w. ∑HBCD and a maximum value of 0.039 ng/g w.w. ∑PBDEs were reported for specimens from English lakes [49]

As regards goldfish, an average value of 2.83 ng/g w.w. ∑HBCD and of up to 0.69 ng/g w.w. for α-HBCD was registered for fishes caught in the Czech Republic [49,51]. Qiu et al. [52] reported for crayfish average values of ∑PBDEs and ∑HBCDs of 0.026 and 0.041 ng/g w.w., respectively, reflecting a contamination level similar to the present study.

As shown, a comparison between studies is likely to result in large variations in BFR concentrations in fish meat, as these largely depend on the pollution pressure at the sampling site, on the year of sampling, on specific metabolic differences and on the age of the fish and lipid content as well as other physiological differences among fish species [53,54].

Furthermore, another reason for the different contamination patterns observed for species inhabiting the same aquatic environment may be found in the different feeding habits of fishes. A previous study found that carnivorous fish species may present higher amounts of contaminants than herbivorous and detritivorous ones, suggesting the potential for biomagnification of BFRs via the trophic chain [55]. In particular, eels are carnivorous benthic feeders and are prone to accumulating pollutants from sediments in addition to other contaminant pathways. 

The dominance, among the other congeners, of BDE-47 shown in the present study is congruent with the general pattern found in fish species in the literature [2,15]; this outcome is likely attributable to the fact that BDE-47 is one of the main components of penta-BDE commercial formulation which was used worldwide [39]. In particular, it has been reported that BDE-47 is a major congener that bioaccumulates in freshwater fish species [56], possibly due to the higher uptake efficiency for BDE-47 from the environment [57], especially for eel and carp [58]. Furthermore, a biotransformation pathway of BDE-99 to BDE-47 in fish tissue has also been hypothesized [59]. Lower amounts of BDE-99 and -100 were observed in the present study in agreement with other studies [39].

In HBCD technical mixtures, the γ- isomer is the major component [11,15]; however, as reported by some authors in animal tissues and in food of animal origin, α-HBCD is usually found to be predominant, followed by γ-HBCD and β- [15,60,61]. The results of the present study are in line with those in the literature [46,49,50,51,52]. The predominance of α-HBCD in biota samples is probably related to the selective metabolism or biotransformation of the three isomers [62]. For instance, Szabo et al. [63] observed in mice the bio-isomerization of γ-HBCD to α-HBCD; in addition, in vitro experiments showed a faster rate of biotransformation of β- and γ-HBCD than α-HBCD [64]. It is also plausible that physicochemical differences among the HBCD molecules can contribute to the mentioned pattern: α-HBCD has a relatively higher water solubility than γ- and β-HBCD, which may result in some preferential uptake of this isomer in the aquatic environment [65]. 

Concerning the average amount of fish consumption, the data reported in the present study are lower than those of the literature [2,66]. EFSA [2] reports a daily fish consumption of 2.6 g/kg b.w. by the European population; however, these data refer to high consumers and to marine and freshwater fish consumption. Malarvannan et al. [39] calculated an average consumption of eel of 0.041 g/b.w./day, in line with what has been reported in the present study considering several fish species. 

Congruent with the data on occurrence and with the literature, the exposure of the general population is evidently highly variable among different parts of the world and also within some individual countries [67]. The values reported in the present study are lower than those reported in the literature [2]. This discrepancy may be due mainly to the lower level of contamination registered for Lake Trasimeno fish species and in part to the contained values of freshwater fish consumption. 

As shown in a previous study on exposure assessment to PBDEs, the highest estimated intake in the present study was from BDE 47 and BDE 209 [2,3,4,5,6,7,8,9,10,11,12,13,14,15,16,17,18,19,20,21,22,23,24,25,26,27,28,29,30,31,32,33,34,35,36,37,38,39,40,41,42,43,44,45,46,47,48,49,50,51,52,53,54,55,56,57,58,59,60,61,62,63,64,65,66,67,68]. In the literature, it is reported that BDE 47 occurred at the highest levels in fish and fish products, and as a consequence, frequent fish consumers are more exposed to higher levels of BDE-47 than the general population [68,69]. 

The observed exposure data and risk characterization according to the EFSA approach resulted in calculated MOE values remarkably higher than the critical values for PBDE-47, -99, -153 and -209, as well as for ∑HBCDs, indicating that that the estimated dietary exposure through consumption of fish from Lake Trasimeno is unlikely to be a significant health concern for the central Italian population. It should be pointed out that the presented risk assessment was performed only for toxicologically relevant congeners and for a specific population subgroup; therefore, the cumulative EDI for the potential presence of various BFRs and their possible metabolites in foodstuff is likely to be under-considered. Zacs et al. [16] defined an MOE lower than that reported in the present study, albeit still below the critical values, ranging from 46 to 1027, for a Latvian population consuming some selected foods of both animal and vegetal origin. Fromme et al. [67] calculated for ∑PBDE an MOE of 240 for infants for total diet in the United States of America. Different exposure scenarios can result in MOEs ranging over several orders of magnitude, and although this makes it difficult to generalize about the risks to health and to compare studies, such exposure scenarios have the potential to be helpful for prioritization and risk management actions.

The comparative assessment between perch samples from the two different lakes revealed higher values of toxicologically relevant contaminants in Piediluco Lake. The reasons for these results probably lie in the fact that this basin is connected to the Rivers Nera and Velino, becoming a reservoir subjected to pollution load due to the discharge of the two rivers, while Trasimeno Lake is characterized by the absence of a tributary and the absence of industry and industrial pressure.

## 5. Conclusions

After the ban on or restricted use of PBDE and HBCD commercial mixtures in the EU, recent exposure to these contaminants is most likely due to the release of such molecules that have accumulated in environmental matrices, such as soils and sediments, and to those still present in manufactured materials. The fact that HBCDs and PBDEs are still found in fish also indicates their high bioavailability and bioaccumulation potential. The level of fish contamination and, consequently, the general population’s exposure to them are characterized by high variability among different areas of the world and even within the same country. Overall, based on currently available toxicological data, it can be concluded that PBDE and HBCD show no health risks for the central Italian population consuming freshwater fish products from Lake Trasimeno. However, for fish species inhabiting an ecosystem characterized by a relevant pollution pressure, an appropriate monitoring plan by means of official control activity is crucial to ensure the health of consumers.

## Figures and Tables

**Table 1:** Average LB and UB values (pg/g) of all investigated PBDE and HBCD congeners for the freshwater species of Lake Trasimeno.

	Perch (n = 16)		Eel (n = 12)		Tench (n = 13)		Goldfish (n = 22)		Crayfish (n = 16)		Carp (n = 11)	
	LB	UB	LB	UB	LB	UB	LB	UB	LB	UB	LB	UB
BDE-28	0.00	10.00	0.00	10.00	0.00	10.00	0.00	10.00	0.71	10.09	0.00	10.00
BDE-49	0.00	10.00	27.68	29.34	0.00	10.00	0.00	10.00	0.66	10.03	0.00	10.00
BDE-47	5.11	10.11	237.13	237.13	0.00	10.00	1.17	10.26	4.89	13.01	1.73	10.82
BDE-66	0.00	10.00	0.00	10.00	0.00	10.00	0.00	10.00	0.94	9.69	0.00	10.00
BDE-77	0.00	10.00	0.00	10.00	0.00	10.00	0.00	10.00	1.08	9.83	0.00	10.00
BDE-100	0.00	10.00	106.92	107.75	0.00	10.00	0.00	10.00	1.33	10.08	0.00	10.00
BDE-99	0.00	10.00	12.54	15.04	0.00	10.00	0.00	10.00	2.60	11.35	0.00	10.00
BDE-85	0.00	10.00	0.00	10.00	0.00	10.00	0.00	10.00	0.67	10.05	0.00	10.00
BDE-154	0.00	10.00	47.61	48.44	0.00	10.00	0.00	10.00	0.62	9.99	0.00	10.00
BDE-153	0.00	10.00	5.28	11.12	0.00	10.00	0.00	10.00	0.65	10.02	0.00	10.00
BDE-138	0.00	10.00	0.00	10.00	0.00	10.00	0.00	10.00	1.38	10.75	0.00	10.00
BDE-183	0.00	10.00	0.00	10.00	0.00	10.00	0.00	10.00	0.00	10.00	0.00	10.00
BDE-197	0.00	10.00	0.00	10.00	0.00	10.00	0.00	10.00	0.63	10.00	0.00	10.00
BDE-206	0.00	100.00	0.00	100.00	0.00	100.00	0.00	100.00	1.85	95.60	0.00	100.00
BDE-209	0.00	100.00	0.00	100.00	0.00	100.00	0.00	100.00	23.22	110.72	0.00	100.00
∑PBDE	5.11	330.11	437.15	718.82	0.00	330.00	0.99	330.26	36.63	341.21	1.73	330.82
∑PBDE (l.w.)	630.86	39,211.11	1797.49	2955.67	0.00	10,576.92	97.06	32,378.43	8721.43	81,240.48	37.94	7254.82
α-HBCD	5.32	10.94	714.81	714.81	1.33	10.56	2.42	10.60	189.93	193.06	11.56	17.92
β-HBCD	0.00	10.00	5.45	14.62	0.00	10.00	0.00	10.00	110.85	115.85	0.00	10.00
γ-HBCD	0.00	10.00	10.41	17.91	0.00	10.00	0.77	10.32	654.57	657.07	0.00	10.00
∑HBCD	4.73	30.94	730.67	747.33	1.24	30.56	2.70	26.17	849.20	965.98	11.56	37.92
∑HBCD (l.w.)	583.95	3665.43	3004.40	3072.90	39.74	979.49	264.71	2565.69	202,190.48	229,995.24	253.51	831.58

LB = lower bound, UB = upper bound, BDE = brominateddiphenyl ether, PBDE = polybrominateddiphenyl ethers, HBCDs = hexabromocyclododecanes.

**Table 2:** Estimated daily intake (pg/kw b.w./day).

	Perch		Eel		Tench		Goldfish		Crayfish		Carp		∑	
	LB	UB	LB	UB	LB	UB	LB	UB	LB	UB	LB	UB	LB	UB
BDE-28	0.000	0.004	0.000	0.000	0.000	0.005	0.000	0.005	0.001	0.005	0.000	0.005	0.000	0.028
BDE-49	0.000	0.004	0.013	0.014	0.000	0.005	0.000	0.005	0.001	0.005	0.000	0.005	0.013	0.037
BDE-47 *	0.002	0.005	0.112	0.112	0.000	0.005	0.001	0.005	0.002	0.006	0.001	0.005	0.118	0.137
BDE-66	0.000	0.004	0.000	0.005	0.000	0.005	0.000	0.005	0.000	0.005	0.000	0.005	0.000	0.028
BDE-77	0.000	0.004	0.000	0.005	0.000	0.005	0.000	0.005	0.001	0.005	0.000	0.005	0.000	0.028
BDE-85	0.000	0.004	0.000	0.005	0.000	0.005	0.000	0.005	0.000	0.005	0.000	0.005	0.000	0.028
BDE-99 *	0.000	0.004	0.006	0.007	0.000	0.005	0.000	0.005	0.001	0.005	0.000	0.005	0.007	0.031
BDE-100	0.000	0.004	0.050	0.051	0.000	0.005	0.000	0.005	0.001	0.005	0.000	0.005	0.051	0.074
BDE-138	0.000	0.004	0.000	0.005	0.000	0.005	0.000	0.005	0.001	0.005	0.000	0.005	0.001	0.028
BDE-153 *	0.000	0.004	0.002	0.005	0.000	0.005	0.000	0.005	0.000	0.005	0.000	0.005	0.003	0.028
BDE-154	0.000	0.004	0.022	0.023	0.000	0.005	0.000	0.005	0.000	0.005	0.000	0.005	0.023	0.046
BDE-183	0.000	0.004	0.000	0.005	0.000	0.005	0.000	0.005	0.000	0.005	0.000	0.005	0.000	0.028
BDE-197	0.000	0.004	0.000	0.005	0.000	0.005	0.000	0.005	0.000	0.005	0.000	0.005	0.000	0.028
BDE-206	0.000	0.044	0.000	0.047	0.000	0.047	0.000	0.047	0.001	0.045	0.000	0.047	0.001	0.278
BDE-209 *	0.000	0.044	0.000	0.047	0.000	0.047	0.000	0.047	0.011	0.052	0.000	0.047	0.011	0.285
∑PBDE	0.002	0.146	0.206	0.339	0.000	0.1556	0.001	0.156	0.019	0.161	0.001	0.156	0.229	1.113
∑PBDE *	0.002	0.058	0.120	0.171	0.000	0.0613	0.001	0.061	0.015	0.068	0.001	0.062	0.138	0.482
														
α-HBCD	0.003	0.005	0.337	0.337	0.001	0.0050	0.0011	0.005	0.089	0.091	0.005	0.008	0.436	0.452
β-HBCD	0.000	0.004	0.003	0.007	0.000	0.0047	0.0000	0.005	0.052	0.055	0.000	0.005	0.055	0.080
γ-HBCD	0.000	0.004	0.005	0.008	0.000	0.0047	0.0004	0.005	0.309	0.310	0.000	0.005	0.314	0.337
∑HBCD *	0.003	0.014	0.344	0.352	0.001	0.0144	0.0015	0.015	0.450	0.455	0.005	0.018	0.805	0.868

LB = lower bound, UB = upper bound, BDE = brominateddiphenyl ether, PBDE = polybrominateddiphenyl ethers, HBCDs = hexabromocyclododecanes, * = toxicologically relevant molecules.

## Data Availability

The datasets generated for this study are available on request from the corresponding author.

## Supplementary Materials

The following are available online at https://www.mdpi.com/article/10.3390/ijerph18168763/s1, Figure S1: Trasimeno Lake ( 43°08′ N 12°06′ E), Figure S2: Lipid content (g/100g); EPA and DHA concentration in fish muscle (mg/100g food), Table S1a: Detailed contamination levels for perch, eel, and tench caught in Trasimeno Lake (pg/g), Table S1b: Detailed contamination levels for goldfish, Crayfish and carp caught in Trasimeno Lake (pg/g), Table S2: Detailed MOE values related to the exposure of consumer s to fish samples from Trasimeno lake, Table S3. Values of benefit-risk quotient related to the consumption of freshwater fish products from Trasimeno Lake (LB = lower bound, UB = upper bound, BDE =brominateddiphenyl ether, ΣHBCD = sum of hexabromocyclododecanes), Table S4. Values (pg/g) of polybrominateddiphenyl ethers (PBDEs) and hexabromocyclododecanes (HBCDs) for perch (*Perca fluviatilis,* n = 10) caught in Piediluco Lake.
