# Supplementary material for: Risk Characterization and Benefit–Risk Assessment of Brominated Flame Retardant in Commercially Exploited Freshwater Fishes and Crayfish of Lake Trasimeno, Italy
Source: Int J Environ Res Public Health. 2021 Aug 19;18(16):8763. doi: 10.3390/ijerph18168763 (PMC8393685; doi:10.3390/ijerph18168763)
Supplement: Supplementary file 1 [file ijerph-18-08763-s001.zip › ijerph-1311300-Supplementary.pdf]

Figure S1. Trasimeno Lake (43°08’N 12°06’E)

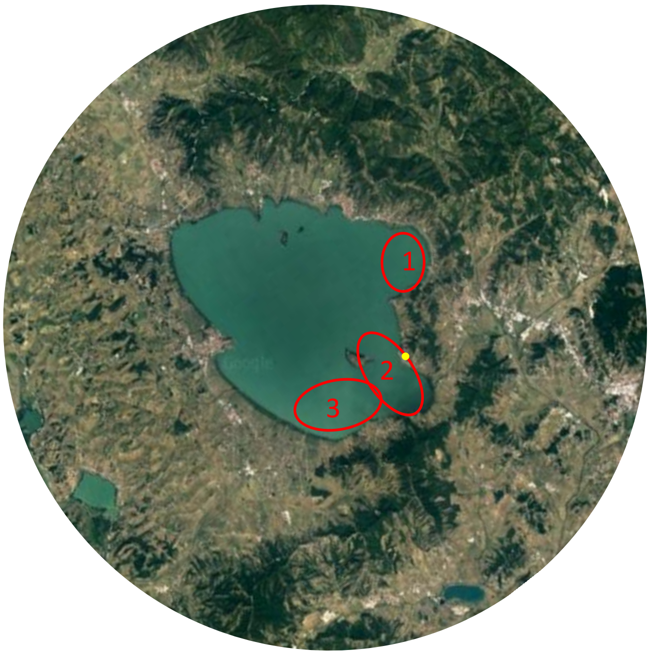

The fishing areas in Trasimeno Lake are highlighted in red. Zone 1= Torricella area. Zone 2= San Feliciano area. Zone 3= Sant’Arcangelo area. The yellow spot refers to the location of Cooperativa dei Pescatori del Lago Trasimeno. San Feliciano.

Figure S2. Lipid content (g/100g); EPA and DHA concentration in fish muscle (mg/100g food)

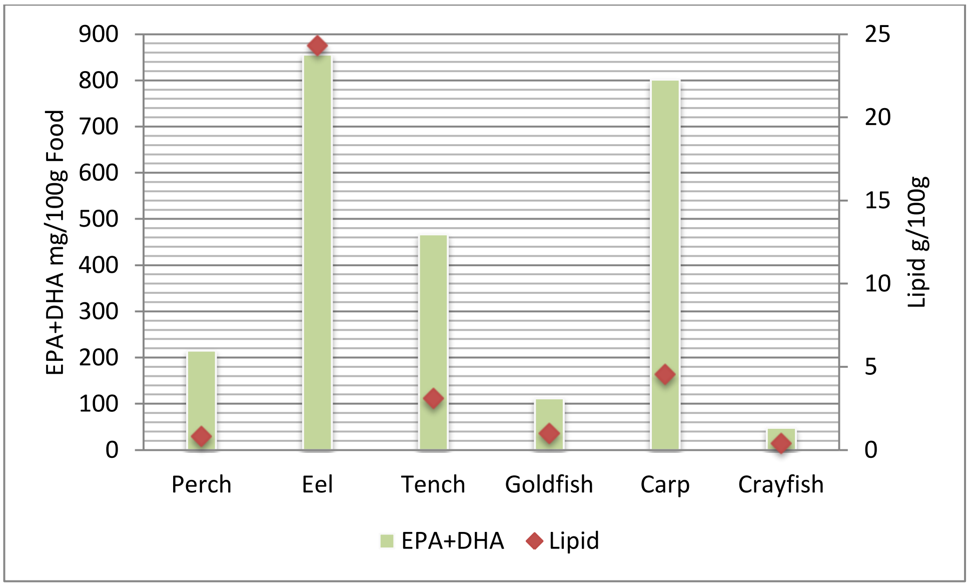

Table S1a. Detailed contamination levels for perch, eel, and tench caught in Trasimeno Lake (pg/g)

	Perch (*n=16*)					Eel (*n=12*)					Tench *(n=13)*				
	average	min	max	LB	UB	average	min	max	LB	UB	average	min	max	LB	UB
BDE-28	n.d.	<10	<10	0.00	10.00	n.d	<10	<10	0.00	10.00	n.d	<10	<10	0.00	10.00
BDE-49	0.00	<10	<10	0.00	10.00	27.68	<10	62	27.68	29.34	0.00	<10	<10	0.00	10.00
BDE-47	5.11	<10	14	5.11	10.11	237.13	46	471	237.13	237.13	1.17	<10	<10	0.00	10.00
BDE-66	n.d	<10	<10	0.00	10.00	n.d	<10	<10	0.00	10.00	n.d	<10	<10	0.00	10.00
BDE-77	n.d	<10	<10	0.00	10.00	n.d	<10	<10	0.00	10.00	n.d	<10	<10	0.00	10.00
BDE-100	n.d	<10	<10	0.00	10.00	106.92	18	536	106.92	107.75	n.d	<10	<10	0.00	10.00
BDE-99	n.d	<10	<10	0.00	10.00	12.54	<10	23	12.54	15.04	n.d	<10	<10	0.00	10.00
BDE-85	n.d	<10	<10	0.00	10.00	n.d	<10	<10	0.00	10.00	n.d	<10	<10	0.00	10.00
BDE-154	n.d	<10	<10	0.00	10.00	47.61	<10	86	47.61	48.44	n.d	<10	<10	0.00	10.00
BDE-153	n.d	<10	<10	0.00	10.00	5.28	<10	16	5.28	11.12	n.d	<10	<10	0.00	10.00
BDE-138	n.d	<10	<10	0.00	10.00	n.d	<10	<10	0.00	10.00	n.d	<10	<10	0.00	10.00
BDE-183	n.d	<10	<10	0.00	10.00	n.d	<10	<10	0.00	10.00	n.d	<10	<10	0.00	10.00
BDE-197	n.d	<10	<10	0.00	10.00	n.d	<10	<10	0.00	10.00	n.d	<10	<10	0.00	10.00
BDE-206	n.d	<100	<100	0.00	100.00	n.d	<100	<100	0.00	100.00	n.d	<100	<100	0.00	100.00
BDE-209	n.d	<100	<100	0.00	100.00	n.d	<100	<100	0.00	100.00	n.d	<100	<100	0.00	100.00
α-HBCD	5.32	<10	18	5.32	10.94	714.81	60	3810	714.81	714.81	2.42	<10	17	1.33	10.56
β-HBCD	n.d	<10	<10	0.00	10.00	5.45	<10	65	5.45	14.62	0.00	<10	<10	0.00	10.00
γ-HBCD	n.d	<10	<10	0.00	10.00	10.41	<10	56	10.41	17.91	0.77	<10	<10	0.00	10.00

LB= lower bound, UB= upper bound, BDE =brominateddiphenyl ether, HBCD= hexabromocyclododecane

Table S1b. Detailed contamination levels for goldfish, Crayfish and carp caught in Trasimeno Lake (pg/g)

	Goldfish (*n=22*)					Crayfish (*n=16*)					Carp *(n=13)*				
	average	min	max	LB	UB	average	min	max	LB	UB	average	min	max	LB	UB
BDE-28	n.d	<10	<10	0.00	10.00	0.71	<10	11.42	0.71	10.09	n.d	<10	<10	0.00	10.00
BDE-49	n.d	<10	<10	0.00	10.00	0.66	<10	10.49	0.66	10.03	n.d	<10	<10	0.00	10.00
BDE-47	1.17	<10	14.6	1.17	10.26	4.89	<10	37.71	4.89	13.01	1.73	<10	19.00	1.73	10.82
BDE-66	n.d	<10	<10	0.00	10.00	0.94	<10	15.08	0.94	9.69	n.d	<10	<10	0.00	10.00
BDE-77	n.d	<10	<10	0.00	10.00	1.08	<10	17.24	1.08	9.83	n.d	<10	<10	0.00	10.00
BDE-100	n.d	<10	<10	0.00	10.00	1.33	<10	11.82	1.33	10.08	n.d	<10	<10	0.00	10.00
BDE-99	n.d	<10	<10	0.00	10.00	2.60	<10	24.86	2.60	11.35	n.d	<10	<10	0.00	10.00
BDE-85	n.d	<10	<10	0.00	10.00	0.67	<10	10.79	0.67	10.05	n.d	<10	<10	0.00	10.00
BDE-154	n.d	<10	<10	0.00	10.00	0.62	<10	9.86	0.62	9.99	n.d	<10	<10	0.00	10.00
BDE-153	n.d	<10	<10	0.00	10.00	0.65	<10	10.34	0.65	10.02	n.d	<10	<10	0.00	10.00
BDE-138	n.d	<10	<10	0.00	10.00	1.38	<10	22.03	1.38	10.75	n.d	<10	<10	0.00	10.00
BDE-183	n.d	<10	<10	0.00	10.00	n.d	<10	0.00	0.00	10.00	n.d	<10	<10	0.00	10.00
BDE-197	n.d	<10	<10	0.00	10.00	0.63	<10	10.04	0.63	10.00	n.d	<10	<10	0.00	10.00
BDE-206	n.d	<100	<100	0.00	100.00	1.85	<100	29.57	1.85	95.60	n.d	<100	<100	0.00	100.00
BDE-209	n.d	<100	<100	0.00	100.00	23.22	<100	253.00	23.22	110.72	n.d	<100	<100	0.00	100.00
α-HBCD	2.42	<10	16.7	2.42	10.60	189.93	<10	1851.55	189.93	193.06	11.56	<10	77.82	11.56	17.92
β-HBCD	n.d	<10	<10	0.00	10.00	110.85	<10	1029.45	110.85	115.85	n.d	<10	<10	0.00	10.00
γ-HBCD	0.77	<10	17	0.77	10.32	654.57	<10	6119.0	654.57	657.07	n.d	<10	<10	0.00	10.00

LB= lower bound, UB= upper bound, BDE =brominateddiphenyl ether, HBCD= hexabromocyclododecane

Table S2. Detailed MOE values related to the exposure of consumer s to fish samples from Trasimeno lake.

	Perch		Eel		Tench		Goldfish		Crayfish		Carp	
	LB	UB	LB	UB	LB	UB	LB	UB	LB	UB	LB	UB
BDE 47	96305521	48676678	2076461	2076461	> 100000000	49212121	420616421	47965030	100638285	37826381	246060606	44738292
BDE 99	> 100000000	2035278	1468531	1272727	> 100000000	1909091	> 100000000	1909091	7342657	1682018	> 100000000	1909091
BDE 153	> 100000000	14020805	26303030	11955923	> 100000000	13151515	> 100000000	13151515	202331002	13125265	> 100000000	13151515
BDE 209	> 100000000	38464646	> 100000000	36060606	> 100000000	36060606	> 100000000	36060606	155299768	32569189	> 100000000	36060606
∑ HBCD	354282786	56441818	2292418	2243317	1351417400	54834999	620650954	64033534	1973337	1734775	139646465	44098884

LB= lower bound, UB= upper bound, MOE= margin of exposure

Table S3. Values of benefit-risk quotient related to the consumption of freshwater fish products from Trasimeno Lake (LB= lower bound, UB= upper bound, BDE =brominateddiphenyl ether, ∑HBCD=sum of hexabromocyclododecanes)

	LB	UB
Perch		
BDE 47	0.000	0.000
BDE 99	<0.00	0.000
BDE 153	<0.00	0.000
BDE 209	<0.00	0.000
∑ HBCD	0.000	0.000
Eel		
BDE 47	0.001	0.001
BDE 99	0.00	0.000
BDE 153	0.00	0.000
BDE 209	<0.00	0.000
∑ HBCD	0.002	0.002
Tench		
BDE 47	<0.00	0.000
BDE 99	<0.00	0.000
BDE 153	<0.00	0.000
BDE 209	<0.00	0.000
∑ HBCD	0.000	0.000
Goldfish		
BDE 47	0.000	0.000
BDE 99	<0.00	0.000
BDE 153	<0.00	0.000
BDE 209	<0.00	0.000
∑ HBCD	0.000	0.000
Crayfish		
BDE 47	0.000	0.001
BDE 99	0.000	0.001
BDE 153	0.000	0.000
BDE 209	0.000	0.000
∑ HBCD	0.030	0.034
Carp		
BDE 47	0.000	0.000
BDE 99	<0.00	0.000
BDE 153	<0.00	0.000
BDE 209	<0.00	0.000
∑ HBCD	0.000	0.000

Table S4. Values (pg/g) of polybrominateddiphenyl ethers (PBDEs) and hexabromocyclododecanes (HBCDs) for perch (*Perca fluviatilis,* n=10) caught in Piediluco Lake.

	average	min	max	LB	UB
BDE28	3.51	<10	35.06	3.51	12.51
BDE49	14.88	<10	74.11	14.88	17.88
BDE47	129.85	48.72	431.64	129.85	129.85
BDE66	1.68	<10	16.77	1.68	10.68
BDE77	0.00	<10	<10	0.00	10.00
BDE100	35.07	18.29	130.62	35.07	35.07
BDE99	77.64	19.07	290.20	77.64	77.64
BDE85	0.00	<10	<10	0.00	10.00
BDE154	26.01	<10	125.67	26.01	27.01
BDE153	12.93	<10	85.94	12.93	18.93
BDE138	0.00	<10	<10	0.00	10.00
BDE183	4.48	<10	44.77	4.48	13.48
BDE197	3.67	<10	36.69	3.67	12.67
BDE206	0.00	<100	<100	0.00	100.00
BDE209	29.76	<100	297.58	29.76	119.76
α-HBCD	747.85	215	35.06	747.85	747.85
β-HBCD	8.26	<10	74.11	8.26	12.26
γ-HBCD	35.93	<10	431.64	35.93	40.93

LB= lower bound, UB= upper bound, BDE =brominateddiphenyl ether, HBCD= hexabromocyclododecane
